# The Relationship of Diet and Physical Activity with Weight Gain and Weight Gain Prevention in Women of Reproductive Age

**DOI:** 10.3390/jcm10112485

**Published:** 2021-06-04

**Authors:** Mamaru Ayenew Awoke, Helen Skouteris, Maureen Makama, Cheryce L. Harrison, Thomas Philip Wycherley, Lisa J. Moran

**Affiliations:** 1 Monash Centre for Health Research and Implementation, Monash University, Clayton, VIC 3168, Australia; mamaru.awoke@monash.edu (M.A.A.); maureen.makama@monash.edu (M.M.); cheryce.harrison@monash.edu (C.L.H.); 2 Health and Social Care Unit, School of Public Health and Preventive Medicine, Monash University, Melbourne, VIC 3004, Australia; helen.skouteris@monash.edu; 3 Alliance for Research in Exercise, Nutrition and Activity, University of South Australia, Adelaide, SA 5001, Australia; Tom.Wycherley@unisa.edu.au

**Keywords:** diet, physical activity, preconception, pregnancy, postpartum, weight gain

## Abstract

Reproductive-age women often see increased weight gain, which carries an increased risk of long-term overweight and obesity and adverse maternal and child health outcomes. Supporting women to achieve optimal weight through lifestyle modification (diet and physical activity) is of critical importance to reduce weight gain across key reproductive life-stages (preconception, pregnancy and postpartum). This review comprehensively summarizes the current state of knowledge on the contribution of diet and physical activity to weight gain and weight gain prevention in reproductive-aged women. Suboptimal diets including a higher proportion of discretionary choices or energy intake from fats, added sugars, sweets or processed foods are associated with higher weight gain, whereas increased consumption of core foods including fruits, vegetables and whole grains and engaging in regular physical activity are associated with reduced weight gain in reproductive age women. Diet and physical activity contributing to excessive gestational weight gain are well documented. However, there is limited research assessing diet and physical activity components associated with weight gain during the preconception and postpartum period. This review highlights the need for further research to identify key dietary and physical activity components targeting the critical windows of reproductive life-stages in women to best guide interventions to prevent weight gain.

## 1. Introduction

Overweight and obesity affect one-third of the world’s population and are associated with a range of chronic health outcomes including type 2 diabetes (DM2), hypertension, cancer, cardiovascular diseases (CVD) and mortality [1,2,3]. The rate of obesity continues to escalate worldwide, exacerbating many causes of premature death and imposing a substantial economic burden [2,4,5]. According to the World Health Organization, 44% of adults aged 18 or over worldwide had overweight or obesity in 2016 [6]. Of these, 39% of men and 40% of women had overweight while 11% of men and 15% of women had obesity [6]. Evidence from longitudinal data shows adults gain on average 0.5 to 0.8 kg per year [7,8,9]. There is now a large body of evidence from over 20 systematic reviews and meta-analyses [10,11,12,13,14,15,16,17,18,19,20,21,22,23,24,25,26,27,28,29,30], published since 2000, of observational and intervention studies assessing diet and physical activity (PA) components associated with weight gain. These report that increased intake of diets characterized by high energy-dense discretionary foods and beverages are associated with weight gain [11,14,17,18,19,21,23,24,25,30] and limited consumption of discretionary foods and beverages, higher consumption of core foods (e.g., fruit, vegetables, whole grain or dietary fiber) and higher diet quality are associated with reduced weight gain in the adult population [10,12,13,15,16,20,22,26,27,28,29,31]. Evidence also consistently reports that moderate-to-vigorous intensity PA (≥150 min/week) is associated with lower weight gain [32,33]. Similarly, in relation to weight gain prevention, there is an abundance of evidence from several systematic reviews and meta-analyses that have synthesized the literature assessing lifestyle interventions targeting the prevention of weight gain in young adults, under 35 years of age [34,35,36], and in the general population [37,38]. A recent systematic review and meta-analysis evaluated trials assessing weight gain prevention in adults and reported that lifestyle (diet and/or physical activity) interventions result in differences in weight (MD −1.15 kg; 95% CI −1.50, −0.80 kg) compared to controls [39].

Whilst the findings above report on the general adult population, we also know that women of reproductive age are at higher risk of longitudinal weight gain and obesity [40]. The Australian Longitudinal Study on Women’s Health reported an average weight gain of 6.3 kg over 10 years [41], with this rate of weight gain greater in women 18–50 years old compared to women aged 50 and over [42]. This confers an increased risk of long-term overweight and obesity. Reproductive life stages in women including preconception, pregnancy and postpartum are critical windows that drive this weight gain and maternal adiposity [43,44]. Nearly 50% of women enter pregnancy with overweight or obesity [45] and 51% gain weight above the Institute of Medicine (IOM) recommendation [46]. Postpartum, women retain an average of 0.5 to 3 kg from each pregnancy [47]. Preconception overweight and obesity are linked to reduced fertility and delayed conception [48]. Overweight and obesity during preconception or pregnancy additionally increase the risk of maternal complications and adverse birth outcomes [49,50]. Evidence consistently shows that higher pre-pregnancy body mass index (BMI) is a strong predictor of excessive gestational and pregnancy complications [51]. Excessive weight gain during pregnancy is also associated with an elevated risk of gestational diabetes, gestational hypertension and preeclampsia, emergency caesarean delivery, hyperglycemia [52,53] and increases the risk of congenital diseases, macrosomia, preterm birth [54], stillbirth [55], future risk of DM2 [56] and CVD [57]. Excessive gestational weight gain (GWG) is a strong predictor of postpartum weight retention [58,59]. A meta-analysis of observational studies reported that women who gained weight above IOM recommendations during pregnancy (singleton pregnancies) retained an additional 3.1 kg and 4.7 kg after 3 and ≥15 years postpartum, respectively, compared to women who gained weight within recommendations [59]. Postpartum weight retention and excessive weight gain after birth are also associated with increased risk of adverse maternal [60,61] and neonatal outcomes [62] during subsequent pregnancies and risk of longer-term chronic maternal conditions including DM2 [63,64] and CVD [65]. Maternal obesity has additional implications on the offspring including childhood obesity [66,67] and cardiometabolic risk factors [68], which may persist into adulthood [50].

Treatment of established obesity through weight loss interventions is intensive, costly and challenging for participants and largely ineffective and unsustainable [69] with only 20% of individuals successful at long-term weight maintenance after weight loss interventions [70]. Prevention of weight gain is therefore paramount to curb the increasing prevalence of overweight and obesity and particularly in reproductive-age women as a population most susceptible to weight gain. The preconception, pregnancy and postpartum periods are key windows of opportunity for weight gain prevention. Lifestyle improvement targeting women at these key life stages to achieve healthy pre-pregnancy BMI, optimal gestational weight gain and reduce postpartum weight retention will not only reduce the risk of maternal overweight and obesity and associated comorbidities but also improve fertility, pregnancy outcomes and the health of the next generation.

Despite the importance of preventing weight gain in reproductive-aged women [71,72], the majority of these women fail to meet population level recommendations for intake of healthy core food group intakes [73,74], consume unhealthy proportions of discretionary choices [75], have inadequate micronutrient intake [76,77], have suboptimal PA [78] and a high frequency of television watching [79,80]. Whilst there is some research that micronutrients (for example B vitamin intake in excess of recommendations) may contribute to overweight/obesity especially in women in developed countries [81,82], this area of research was not within the scope of the review. Lifestyle changes including optimizing core food groups of fruits, vegetables, whole grains, protein and dairy or alternatives, minimizing intake of discretionary or non-core foods, which are commonly energy-dense or nutritionally poor, and engaging in regular PA will aid in preventing weight gain. The relationship of diet and PA with weight gain and lifestyle interventions to prevent weight gain has been extensively researched in the general population. However, it is not clear what key diet and PA components contribute to weight gain in reproductive-age women at the key life stages of preconception, pregnancy and postpartum that can assist in tailoring future interventions. Hence, the purpose of this narrative review was to synthesize the literature exploring associations of diet and PA with weight gain from both epidemiologic studies and lifestyle interventions to prevent weight gain and aid in the identification of key components to target in the reproductive-life stages of women. Our narrative review compliments and extends the systematic reviews conducted to date for the general adult population by focusing solely on reproductive-aged women, and reporting for the first time on this cohort as well as on the specific life phases of preconception, pregnancy and postpartum; the definitions of these life phases are provided below. Additionally, this review identifies current gaps within this evidence base and informs directions for future research.

## 2. The Association of Diet and Physical Activity with Weight

### 2.1. Diet, Physical Acitvity and Wieght Gain in Reproductive-Aged Women

A summary of studies assessing the association of diet and PA with weight gain in reproductive-aged women (aged 18–50 years) are shown in Table 1. The studies were sourced from the following databases: Ovid Medline, EMBASE, CINAHL Plus, PsycINFO and all EBM reviews using the following search terms: “weight gain/or weight adj2 (gain or change) or bmi adj2 (gain or change) or (body mass index) adj2 (gain or change) AND diet/or diet therapy/or diet* or nutri* or nutrition/or physical activity or exercise AND women/or women’s health/or women or woman”. Papers published from 2000 onwards were included if they focused on diet and/or PA and weight gain in women of reproductive age. Only studies published in English were included.

#### 2.1.1. Energy Density and Discretionary Choices

A longitudinal study (*n* = 186) reported that women (aged 24–46 years) who consume diets with higher energy density (≥1.85 kcal/g) gained more weight than women consuming diets with lower energy density (≤1.5 kcal/g) (6.4 kg vs. 2.5 kg) after six years [83]. In this study, women who had lower energy density diets reported significantly lower total energy intakes and consumed fewer servings of baked desserts, refined grains and fried vegetables and more servings of vegetables, fruit, and cereal compared with women who had higher energy density diets. A similar study in a large cohort of women (aged 22–44 years, *n* = 50,026) followed up for 8 years reported that women who had higher dietary energy density (5th quintile) gained more weight than women who had lower dietary energy density (1st quintile) (6.4 kg vs. 4.6 kg) [84]. Here, a higher energy density represented high intake of saturated and trans fats and refined carbohydrate and lower intake of vegetables and fruits [84]. Weight gain also varied considerably according to the energy density of individual foods and beverages. Consumption of some foods/beverages with low energy density such as soda, potatoes and fruit punches were associated with greater weight gain whereas consumption of some foods with relatively higher energy density such as olive oil and nuts were not associated with weight gain [84]. This implies that selecting foods based on energy density alone can be misleading and that consideration of the status of foods and beverages as core or discretionary also must be considered. Findings from observational studies suggest that increased consumption of sugar sweetened beverages (SSBs) and fruit juices is associated with weight gain in reproductive-aged women. Schulze et al. [85] reported that women (age 22–44 years, *n* = 51,603) who increased their intake of SSBs from ≤1 drink/week to ≥1 drinks/day over two 4-year periods (1991–1995 and 1995–1999) gained weight (4.7 kg and 4.2 kg, respectively). Conversely, women who decreased SSBs intake from ≥1 drink/day to ≤1 drink/week gained 1.5 kg and 0.14 kg, respectively, over the same periods [85]. Similarly, women with increased consumption of fruit juice (≤1 drink/week in 1991 to ≥1 drink/day in 1995) gained more weight compared to those with decreased consumption (≥1 drink/day in 1991 to ≤1 drink/week in 1995) (4.03 kg vs. 2.34 kg, *p* < 0.001). In the limited research examining fast food intake and weight gain in reproductive-aged women (20–45 years, *n* = 891), a higher frequency of fast-food restaurant use was associated with an increased total energy intake, percentage energy from fat, hamburgers, French fries and soft drinks and weight. On average, an increase of one fast food meal per week was associated with an increase in average energy intake of 234.4 kJ/day and weight gain of 0.72 kg in women over 3 years [86]. It has been reported that the Western dietary pattern (characterized by high intakes of refined grains, red and processed meats, sweets and potatoes) is associated with weight gain. In a prospective study, women (26–46 years, *n* = 51,670) who increased their Western dietary pattern score (low to high quintiles) had increased weight gain (4.6 kg for 1991 to 1995 and 2.9 kg for 1995 to 1999) compared to women who decreased (high to low quintiles) (2.7 kg for 1991 to 1995 and 1.4 kg for 1995 to 1999) [87]. Data from a prospective study that followed women (30–55 years at baseline, *n* = 41,518) for 8 years reported that the percentage of calories from animal fat, saturated and trans-fat, but not monounsaturated or polyunsaturated fat, was also associated with weight gain (1.04 kg; 95% CI 0.81, 1.29) [88], with a stronger association in overweight women.

There are limited observational studies that assess the association between alcohol consumption and weight gain in reproductive-age women. Wannamethee et al. [89] reported that heavy drinking (>30 g/day of alcohol (>3 standard drinks)), but not light to moderate drinking (0.1 to 29.9 g/day), was associated with higher odds of weight gain (≥5 kg over 8 years) (OR 1.64; 95% CI 1.03, 2.61) compared with non-drinkers in women aged 27–44 years (*n* = 49,324). Similarly, normal weight women (age 38 years and above, *n* = 19,220) who consumed a light to moderate amount of alcohol had less risk of having overweight or obesity compared with non-drinkers over 12.9 years [90]. Conversely, an inverse association was reported between alcohol consumption and weight gain with women aged 38 years and above who did not consume alcohol having higher weight gain (3.6 kg; 95% CI 3.45, 3.80) compared with those who consumed 30 g/day (1.6 kg; 95% CI 0.93, 2.18) during 12.9 years follow-up [90].

#### 2.1.2. Core Foods

A number of studies have reported inverse associations between fruit and vegetable intake and weight gain in women [91,92,93] although many of these studies were not conducted solely in reproductive-age women. For example, Vioque et al. [93] reported that higher intake of fruits (3rd quartile, 249 to 386 g/day compared to 1st quintile, <149 g/day) and vegetables (4th quartile, >333 g/day compared to 1st quintile, <166 g/day) were associated with a reduced risk of weight gain (≥3.4 kg over 10 years) (OR 0.31; 95% CI 0.11, 0.85 and OR 0.18; 95% CI 0.05, 0.66, respectively) in women aged 15–80 years (*n* = 206). An increased intake of both fruits and vegetables was also associated with a lower risk of weight gain, with the lowest risk in the fourth quartile (<362 g/day vs. >698 g/day) (OR 0.22; 95% CI 0.06, 0.81). It has also been reported that adequate intake of fruit and vegetables helps to maintain weight in women of reproductive age with overweight or obesity. A cross-sectional study reported that women with overweight/obesity (age 18–49 years, *n* = 279) consuming >5 fruits and vegetables servings/day were 94% more likely to be weight resilient (maintaining a normal weight in a food desert environment) (prevalence ratio 1.94; 95% CI 1.10, 3.43) compared to those consuming <5 servings/day [94]. A “prudent” dietary pattern characterized by high intakes of fruits, vegetables and whole grains was also associated with less weight gain. Women (26–46 years) with increased “prudent” pattern score gained less weight (1.9 kg for 1991 to 1995 and 0.7 kg for 1995 to 1999) than women with decreased “prudent” pattern score (4.8 and 3.4 kg in the two time periods) [87].

#### 2.1.3. Diet Quality

Diet quality scores represent adherence to population-based dietary guidelines with higher scores generally associated with improved compliance to guidelines [95,96]. A longitudinal study in young women (29–37 years, *n* = 4287) reported that overall diet quality measured by the Australian Recommended Food Score (ARFS) and fruit and vegetable index (FAVI) was associated with lower weight gain. Young women in the highest tertile of FAVI and ARFS had less weight gain compared with the lowest tertile over 6 years (−1.6 kg; 95% CI −2.4, −0.3) and (−1.6 kg; 95% CI −2.67, −0.56), respectively [97]. Similar recent longitudinal studies also reported that higher diet quality assessed by ARFS or FAVI was associated with less weight gain in young women (aged 27–31 years, *n* = 4083) over 6 years [98].

#### 2.1.4. Physical Activity

As reported in the general population, there is also evidence of an inverse association between PA and weight gain in women of reproductive age. In a longitudinal study over 8 years in 46,754 healthy women (aged 25–43 years), Mekary et al. assessed time spent in moderate to vigorous exercise (e.g., hiking, jogging, running, bicycling, aerobics/aerobic dance/rowing machine, swimming and weight gain (classified as more than a 5% change) [99]. They found that women who sustained ≥30 min/day of total PA were less likely to gain weight (OR 0.68; 95% CI 0.64, 0.73) compared with women who sustained <30 min/day. A dose–response relationship was observed such that increased duration of daily PA was associated with less weight gain; even an 11–20 min/day increase in PA was found to be beneficial (OR 0.75; 95% CI 0.68, 0.83). Similarly, a 16 year follow up study by Lusk et al. reported that spending 30 min/day in a range of moderate intensity activities, e.g., brisk walking (−1.8 kg; 95% CI −2.05, −1.56) or bicycling (−1.6 kg; 95% CI −2.09, −1.08), was associated with reduced weight gain in premenopausal women (aged 25–45 years, *n* = 18,414).

In summary, there is evidence on weight gain and diet in reproductive-aged women. However, most studies were conducted in developed countries like the US and Australia and in specific cohorts such as the Nurses’ Health study and the Australian Longitudinal Study on Women Health, which makes it difficult to generalize findings to other women of reproductive age worldwide. Overall, findings suggest that increased consumption of discretionary foods or Western diets characterized by high energy-dense foods including highly processed foods or fast food, refined carbohydrates and lower fiber foods may contribute to increased weight gain over time in women of reproductive age. Conversely, increased core food intake including fruits and vegetables and a high fiber diet are inversely associated with weight gain. Insufficient or low levels of PA contributes to long-term weight gain.

### 2.2. Diet, Physical Acitvity and Weight Gain during Pregnancy

The 2009 IOM guidelines recommend an optimal range of GWG based on pre-pregnancy BMI. For a singleton pregnancy this is 12.7–18.1 kg, 11.3–15.9 kg, 6.8–11.3 and 5.0–9.1 kg for women with underweight, normal weight, overweight and obesity, respectively [45]. A summary of studies assessing diet, PA and weight gain during pregnancy are shown in Table 2. The studies were sourced from the following databases: Ovid Medline, EMBASE, CINAHL Plus, PsycINFO and all EBM reviews using the following search terms: “gestational weight gain/or weight adj2 (excess gain or change) or bmi adj2 (gain or change) or (body mass index) adj2 (gain or change) AND diet/or diet therapy/or diet* or nutri* or nutrition/or physical activity or exercise AND pregnancy/or pregnant women or woman”. Papers published from 2000 onwards were included if they focused on diet and/or PA and excessive GWG during pregnancy. Only studies published in English were included.

**Table 1:** Summary of studies that assessed diet, physical activity and weight gain in reproductive-age women.

Reference	Study Design	Study/Participant Information	Dietary and PA Factors Examined	Key Findings
Savage 2008 [83]	Prospective cohort study	Age: 24–46 yrs. Follow-up: 6 yrs. *n* = 186	Dietary ED (kcal/g), calculated from the energy content of all foods (excluding beverages)	Women consuming higher ED (≥1.85 kcal/g) gained more weight than women consuming lower ED (ED ≤ 1.5 kcal/g) (6.4 kg vs. 2.5 kg).
Bes-Rastrollo 2008 [84]	Prospective cohort study (The Nurses’ Health Study II)	Age: 24–44 yrs. Follow-up: 8 yrs. *n* = 50,026	Dietary ED (kcal/g), calculated from the energy content of all foods (without and with excluding beverages)	Increased total dietary ED was associated with a greater weight gain. Women who had higher ED (5th quintile, 1.46 kcal/g) gained more weight than women who had lower dietary ED (1st quintile, 0.83 kcal/g) (6.4 kg vs. 4.6 kg).
Field 2007 [88]	Prospective cohort study (The Nurses’ Health Study)	Age: 30–55 yrs Follow-up: 8 yrs. *n* = 41,518	Dietary fats; percentage of calorie from monounsaturated, polyunsaturated, animal, saturated and trans fats	Percentage of calories from animal, saturated and trans-fats was strongly associated with weight gain. For every percentage increase in calories from trans-fat, there was an average weight gain of 1.04 kg (95% CI 0.81, 1.29).
Schulze 2004 [85]	Prospective cohort study (The Nurses’ Health Study II)	Age: 24–44 yrs. Follow-up: 8 yrs. *n* = 51,603	Consumption of SSBs, soft drinks	Women with increased intake of SSBs from ≤1 drink/week to ≥1drinks/day increased weight (4.7 kg during 1991–5 and 4.2 kg during 1995–9). Women who decreased SSBs intake from ≥1 drink/day to ≤1 drink/week decreased weight (1.5 kg during 1991–5 and 0.14 kg 1995–9). Increased consumption of fruit juice from ≤1 drink/week to ≥1 drink/day increased weight (4.0 kg during 1991–5 vs. 2.3 kg during 1995–9).
Schulze 2006 [87]	Prospective cohort study (The Nurses’ Health Study II)	Age: 26–46 yrs. Follow-up: 8 yrs. *n* = 51,670	Dietary patterns	Women who had an increased Western pattern score gained more weight gain (4.6 kg for 1991 to 1995 and 2.9 kg for 1995 to 1999) than women who had lower Western pattern score (2.7 and 1.4 kg for the two time periods). Women who had an increased Prudent pattern score, characterized by high intakes of fruits, vegetables and whole grains, gained less weight (1.9 kg for 1991 to 1995 and 0.7 kg for 1995 to 1999) compared to women with decreased Prudent pattern score (4.8 and 3.4 kg for the two time periods).
French 2000 [86]	Prospective cohort study (The Nurses’ Health Study II)	Age: 26–46 yrs. Follow-up: 3 yrs. *n* = 891	Dietary intake, fast food restaurant use	A higher frequency of fast-food restaurant use was associated with an increased total energy intake, percentage energy from fat, hamburgers, French fries and soft drinks and weight. On average, an increase of 1 fast food meal per week was associated with an increase of 234.4 kJ/day and weight gain of 0.72 kg in women.
Boggs 2011 [91]	Prospective cohort study	Age: 21–54 yrs. Follow-up: 14 yrs. *n* = 41,351	Dietary patterns	Vegetables/fruit consumption pattern was associated with significantly less weight gain (10.9 and 11.9 kg in the highest and lowest quintiles). Meat/fried foods pattern was associated with significantly greater weight gain (12.0 and 10.2 kg in the highest and lowest quintiles).
Vioque 2008 [93]	Prospective cohort study	Age: 15–80 yrs. Follow-up: 10 yrs. *n* = 206	Fruit and vegetable intake	Compared to women who were in the lowest quartile of fruit consumption (<149 g/day), women in the third quartile (249–386 g/day) reduced their risk of gaining ≥3.41 kg weight by 69% (OR 0.31; 95% CI 0.11, 0.85). Higher vegetable intake also reduced the risk of weight gain in women of the fourth quartile (>333 g/day) (OR 0.18; 95% CI 0.05–0.66). The combined intake of fruits and vegetables decreased the risk of weight gain across quartiles, with the lowest risk among those in the fourth quartile (>698 g/day) (OR 0.22; 95% CI 0.06, 0.81) compared to those in the first quartile (<362 g/day).
Aljadani 2013 [97]	Prospective cohort study	Age: 29–37 yrs. Follow-up: 6 yrs. *n* = 4287	Diet quality assessed by FAVI and ARFS	Women in the highest tertile of FAVI had less weight gain (−1.6 kg; 95% CI −2.4, −0.3). Similarly, women in the highest tertile of the ARFS had less weight gain compared with the lowest tertile (−1.6 kg; 95% CI −2.67, −0.56).
Aljadani 2020 [98]	Prospective cohort study	Age: 27–31 yrs. Follow-up: 6 yrs. *n* = 4083	Diet quality assessed by FAVI and ARFS	Every one-point increase in either the ARFS or FAVI score was associated with significantly less weight gain, 33 and 12 g, respectively. Women in the highest ARFS tertile gained significantly less weight compared with women in the lowest ARFS tertile (3.7 kg vs. 4.1 kg).
Wannamethee 2004 [89]	Prospective cohort study (The Nurses Health Study II)	Age: 27–44 yrs. Follow-up: 8 yrs. *n* = 49,324	Alcohol consumption	Heavy drinking (>30 g/day) was associated with higher odds of weight gain (≥5 kg) (OR 1.6; 5% CI 1.03, 2.61) compared with non-drinkers, particularly in younger women, but light to moderate drinking (<30 g/d) was not associated with weight gain.
Wang 2010 [90]	Prospective cohort study	Age: 38 yrs and above. Follow-up: 12.9 yrs. *n* = 19,220	Alcohol consumption	Women who did not consume alcohol had higher weight gain (3.6 kg; 95% CI 3.45, 3.80) compared with those who consumed 30 g/day (1.6 kg; 95% CI 0.93, 2.18). Normal-weight women who consumed a light to moderate amount of alcohol gained less weight and had a lower risk of overweight/obesitycompared with non-drinkers (0 to less than 5, 5 to less than 15, 15 to less than 30, and ≥30 g/day: ((RR 0.96; 95% CI 0.91, 1.01), (RR 0.86; 95% CI 0.80, 0.92), (RR 0.70; 95% CI 0.62, 0.79) and (RR 0.73; 95% CI 0.62, 0.85), respectively)).
Mekary 2009 [99]	Prospective cohort study (The Nurses’ Health Study II)	Age 25–43 yrs. Follow-up: 8 yrs. *n* = 46,754	PA: activities such as walking or hiking, jogging running, bicycling, aerobics, swimming	Women who sustained ≥30 min/day of total PA were less likely to gain weight (5% change) (OR 0.68; 95% CI 0.64, 0.73) compared with women who sustained <30 min/day.
Lusk 2010 [100]	Prospective cohort study (The Nurses’ Health Study II)	Age: 25–45 yrs. Follow-up: 16 yrs. *n* = 18,414	PA: bicycle riding, brisk walking, time spent during these activities (minutes/day)	Bicycle riding and brisk walking were associated with less weight gain with an inverse dose–response relationship. For a 30 min/day increase in activity, weight gain was significantly less for brisk walking (−1.8 kg; 95% CI −2.05, −1.56) and bicycling (−1.6 kg; 95% CI −2.09, −1.08).

Abbreviations: ARFS = Australia Recommended Food Score; BMI = Body Mass Index; CI = confidence interval; ED = dietary energy density; FAVI = Fruit and Vegetable Index; OR = odd ratio; PA = physical activity; RCTs = randomized controlled trials; SSBs = sugar-sweetened beverages; yrs = years.

#### 2.2.1. Discretionary Choices

Systematic reviews of observational studies consistently report that excessive GWG is positively associated with higher energy intake [101,102]. Several observational studies evaluated the effect of higher intake of added sugar-containing foods and GWG [103,104,105]. A large prospective cohort study of 46,262 Danish women with singleton pregnancies reported that women with an intake of added sugar in the highest quintile (89 g/day) had a higher rate of GWG (additional 34 g/week; 95% CI 28, 40) compared to women in the lowest quintile (19 g/day) and overall gained an extra 1.4 kg during pregnancy [103]. The study further reported that the intake of confectionary (primarily chocolate and mixed candy) was directly associated with GWG (additional 51 g/week; 95% CI 45, 58) [103]. A study that analyzed data from 495 pregnant women reported that women who consume more confectionary in early pregnancy (11–15 weeks of gestation) had increased risk of excess GWG (OR 2.52; 95% CI 1.10, 5.77) [104]. Similarly, women who had a higher intake of added sugar containing foods (≥2/day) gained more weight (5.4 kg; 95% CI 2.1, 8.7) compared with women who had a lower (<1/week) intake [105]. Unhealthy dietary patterns such as higher consumption of fast foods [106], ultra-processed foods [107], Western dietary patterns characterized by energy-dense, processed, high sugar and fat foods [108,109] and fried foods [110] during pregnancy are also associated with excessive GWG.

#### 2.2.2. Core Foods

An inverse association between fruit and vegetable intake and GWG has been reported in some [111,112] but not all [113] studies. A prospective cohort study in pregnant women (*n* = 622) reported a significantly lower GWG among those who had ≥3 compared with <3 servings/day of fruits and vegetables [112]. Consuming increasing daily cup equivalents of fruits and vegetables during pregnancy also reduced the risk of excessive GWG (OR 0.77; 95% CI 0.60, 0.97) [111].

Dietary patterns high in dairy products, fruits, vegetables, and nuts and seeds during pregnancy reduce excessive GWG [114]. Furthermore, healthy dietary patterns including “New Nordic Diet”, “prudent” and the Mediterranean diet have been reported to be associated with lower risk of excessive GWG. A prospective cohort study assessed the New Nordic Diet (NND), characterized by higher consumption of fruits and vegetables, whole grains, potatoes, fish, game, seafood, milk and drinking water, in 66,597 pregnant women and reported that women with normal weight with high adherence to NND had significantly lower odds of excessive GWG (OR 0.93; 95% CI 0.87, 0.99) compared to women with low adherence [115]. In a retrospective cohort study, women (*n* = 1432) with low Mediterranean diet adherence were twice at risk of excessive GWG (OR 1.9; 95% CI 1.52, 2.37) [116].

Observational studies have reported conflicting findings on the relationship between macronutrients and GWG. While a systematic review of observational studies reported that GWG was positively associated with increased total energy intake, the association of specific macronutrient intake and GWG was inconsistent [102]. Several prospective studies reported no association between total fat or saturated fat intake and excessive GWG [114,117,118]. In contrast, a study in Pakistan in 157 women reported that higher total fat intake was associated with higher GWG [119]. A cross-sectional analysis of 224 pregnant women in the US reported a positive association between GWG and animal fat but not vegetable fat intake [120]. With regard to protein and excessive GWG, studies have reported either no [106,118], inverse [121] or positive associations [120]. With regard to carbohydrate, women with high carbohydrate intake (430–629 g/day) during the second trimester had greater GWG (2.3 kg; 95% CI 0.43, 4.08) [122] than women with low carbohydrate intake (229–429 g/day). A recent study reported that consumption of lower carbohydrate diet (% kcal < 49.6%) was associated with less GWG in women with obesity compared to a higher carbohydrate diet (% kcal > 49.6%) (7.9 vs. 13.1 kg), but an opposite pattern was reported in women with normal weight (16.6 vs. 12.9 kg) [123]. Conversely, carbohydrate intake at 27 weeks of gestation was associated with reduced GWG in all BMI groups [120], supported by a systematic review of observational studies [101]. A study by Rugină et al. [121], however, reported no significant association between a carbohydrate-based diet and GWG.

#### 2.2.3. Diet Quality

Studies assessing the association between diet quality and GWG are limited. A cross-sectional study measured diet quality by the 2005 Healthy Eating Index (HEI-2005), reflecting adherence to the MyPyramid US dietary guidelines, and reported no associations between the HEI-2005 and excessive GWG after adjustment for several potential confounders [114]. However, in a prospective cohort study by Yong et al. [124] higher diet quality assessed by the modified Healthy Eating Index for Malaysians (HEI) (reflecting compliance with recommendations for intake of cereals and grains, vegetables, fruits, dairy or alternatives and meat, egg and legumes) in the second and third trimesters were significantly associated with reduced risk of excessive GWG in women with normal weight [124]. A recent study in Sweden evaluated diet quality and its effect on GWG using the National Food Agency’s index which was designed to assess compliance with dietary recommendations of intakes of fruit, vegetables, fish and whole-grain breads [125]. A low-quality diet score was associated with higher odds of excessive GWG compared with a high-quality diet score (reflecting higher intakes of fruits and vegetables, whole grain bread, fish and shellfish and lower intakes of discretionary choices (sweets, cakes, soft drinks and French fries) [125].

#### 2.2.4. Physical Activity

PA declines during pregnancy [126] and this is independently associated with a greater GWG [113,127], regardless of pre-pregnancy BMI [113]. PA therefore plays a significant role in promoting optimal weight gain during pregnancy. Several observational studies consistently report that higher levels of PA during pregnancy are associated with reduced risk of excessive GWG [118,128,129,130,131,132]. PA, including total, walking and vigorous activity, was inversely associated with excessive GWG in a large prospective cohort study of 1388 pregnant women. Specifically, 30 min walking per day (OR 0.92; 95% CI 0.83, 1.01), 30 min vigorous PA per day (OR 0.76; 95% CI 0.60, 0.97) and 30 min per day total PA (OR 0.95; 95% CI 0.89, 1.01) were associated with lower excessive GWG during mid-pregnancy [118]. Conversely, Ehrlich et al. [128] (*n* = 1055) reported that only vigorous-intensity PA was associated with reduced odds of excess GWG (OR 0.63; 95% CI 0.40–0.99) and in women with overweight/obesity (OR 0.46; 95% CI 0.27, 0.79) with no associations reported for moderate-intensity exercise [128]. A prospective cohort study evaluated the role of PA and excessive GWG in 2767 pregnant women and reported that women engaging in ≥150 min/week had reduced odds of GWG exceeding the IOM recommendations (OR 0.71; 95% CI 0.57, 0.88) compared with women with low level of PA (<60 min/week) [132].

In summary, while increased energy intake during pregnancy contributes to excessive GWG, evidence on the contribution of macronutrients is inconsistent. Unhealthy dietary patterns characterized by high saturated fat, refined grains, added sugars and low fiber foods may contribute to excessive GWG. Conversely, increased consumption of core food groups including fruits, vegetables and whole grains, high-quality diet and reducing consumption of discretionary choices may promote optimal GWG and lower risk of excessive GWG. There is sufficient evidence that moderate to vigorous PA during pregnancy is associated with reduced excessive GWG. However, there are limited data specifically on the amount of PA required to prevent excessive GWG.

**Table 2:** Summary of studies assessed diet, physical activity and weight gain during pregnancy.

Reference	Study Design	Study Participant ‡ *n*	Dietary and PA Factors Examined	Key Findings
Tielemans 2016 [102]	Systematic review	56 studies (46 observational studies; 47 longitudinal and 1 case–control study; 10 intervention studies (2 RCTs))	Energy and macronutrients (carbohydrate, protein and fat) intake	Longitudinal studies: Higher energy intake during pregnancy associated with excessive GWG (20/42 studies reported an association between energy intake and GWG). Intervention studies: 9/10 studies reported an association between energy intake and GWG. The association between macronutrient intake and excessive GWG was inconsistent.
Streuling 2011 [101]	Systematic review	12 cohort studies	Energy intake, protein intake	Energy intake was associated with GWG (7/12 studies). Intake of calories from protein, carbohydrate and vegetables was associated with reduced GWG.
Pathirathna 2017 [122]	Prospective cohort study	*n* = 141	Carbohydrate intake	Women with moderate carbohydrate intake (430–629 g/day) gained higher GWG than women with lower carbohydrate intake (229–429 g/day) (2.3 kg; 95% CI 0.43, 4.08) in the second trimester.
Wrottesley 2017 [108]	Prospective cohort study	*n* = 538	Dietary patterns	Traditional diet pattern (characterized by high intake of whole grains, legumes, vegetables and traditional meats and decreased intake of refined, high sugar and fat foods) was associated with reduced excessive GWG (OR 0.81; 95% CI 0.69, 0.94).
Shin 2016 [133]	Cross-sectional study	*n* = 391	Dietary patterns	Women in the mid tertile of Mixed pattern score (characterized by high loadings of added sugar, butter, cheese, cold breakfast cereals, cured meat, dairy products, fruit drinks, fruits, vegetables, high-energy drinks, legumes, nuts and seed, pizza, potatoes, poultry, refined and whole grains) had lower odds of excessive GWG (OR 0.39; 95 % CI 0.15, 0.99).
Renault 2015 [105]	RCT	*n* = 342	Dietary composition, Adherence to a hypocaloric diet (5000–7000 kJ), low in saturated fat, Mediterranean-style	Foods that contributed to the intake of added sugars including confectionary, snacks, cakes, and soft drinks were associated with weight gain. Higher intake of confectionaries (≥2/day) was associated with more weight gain than low confectionary intake (<1/week) intake (5.4 kg; 95% CI 2.1, 8.7) over the entire pregnancy.
Lai 2019 [134]	Prospective cohort study	*n* = 960	Energy intake, macronutrients, food groups	Higher energy intake (per 500 kcal increment) was associated with greater GWG z-scores (0.18; 95% CI 0.13, 0.23). Substitution of carbohydrate for fat (per 5% energy substitution) were associated with greater GWG (0.07; 95% 0.03, 0.12). Higher intakes (3rd tertile) of plant-based protein foods were associated with a lower risk of excessive GWG compared to lower intake (1st tertile) (RR 0.66; 95% CI 0.46, 0.94).
Maugeri 2019 [109]	Prospective cohort study	*n* = 232	Dietary patterns	An increase in Western dietary pattern score (from 1st to 3rd tertile) (characterized by a high intake of red meat, fries, dipping sauces and salty snacks) was associated with increased GWG (1.2 kg, *p* = 0.013).
Maslova 2015 [103]	Prospective cohort study	*n* = 46,262	Macronutrients, protein-to-carbohydrate ratio, added sugar	Highest quintile (1.4 z-score) of protein to carbohydrate ratio had a lower rate of GWG (−16 g/day; 95% CI −22, −9.0) compared to women with the lowest quintile (−1.3 z-score). Women with an intake of added sugar in the highest quintile (89 g/day) compared to the lowest quintile (19 g/day) had a higher rate of weekly weight gain (34 g/week; 95% CI 28–40 g/week).
Augustin 2020 [125]	Prospective cohort study	*n* = 2125	Dietary quality score (score 0–12) assessed by the National Food Agency’s index designed to assess fibre, fat and discretionary choicess	Poor dietary quality (score ≤ 4) was associated with higher odds of excessive GWG (OR 4.4, *p* = 0.01) compared with a high-quality diet score (score ≥ 9).
Lagiou 2004 [120]	Prospective cohort study	*n* = 224	Energy intake, macronutrients	Weight gain at the end of the second trimester was positively associated with energy intake (kJ/day) (0.9 kg/SD, *p* = 0.006) as well as energy-adjusted intakes of protein and lipids of animal origin (3.1 kg/SD, *p* < 0.001; 2.6 kg/SD, *p* < 0.001, respectively) but negatively associated with energy-adjusted intakes of carbohydrates (−5.2 kg/SD, *p* < 0.001).
Uusitalo 2009 [106]	Retrospective cohort study	*n* = 3360	Dietary patterns, food groups	Fast food dietary pattern was associated with higher rate of weight gain (0.01 kg/week). The rate of weight gain was significantly associated with energy intake (0.016 kg/week), % energy from protein (−0.004 kg/week), % energy from saturated fatty acid (0.002 kg/year) and % energy from sucrose (0.002 kg/week).
Stuebe 2009 [118]	Prospective cohort study	*n* = 1388	Energy intake, food groups and types including fried foods, dairy, fruits and vegetables, red and processed meats, whole grains	Total energy intake 500 kcal/day (OR 1.10; 95% CI 1.00, 1.22), total dairy intake serving/day (OR 1.08; 95% CI 1.00, 1.17) and fried foods serving/day (OR 3.47; 95% CI 0.91, 13.24) were associated with excessive GWG. Vegetarian diet in the first trimester was inversely associated with excessive GWG (OR 0.45; 95% CI 0.27, 0.76).
Deierlein 2008 [135]	Prospective cohort study	*n* = 1231	Dietary energy density, energy intake, carbohydrates	Compared with women in the 1st quartile of mean dietary energy density (0.71 kcal/g), women in the 3rd quartile (0.98 kcal/g) and 4th quartile (1.21 kcal/g) gained an excess total GWG (1.13 kg; 95% CI 0.24, 2.01), (1.08 kg; 95% CI 0.20, 1.97), respectively.
Hillesund 2014 [115]	Prospective cohort study	*n* = 66,597	Diet quality assessed by NND (score 0–10)	High adherence to NND (score 6–10) (representing intake of fruits, vegetables, whole grains, potatoes, fish, game, milk) was associated with lower odds of excessive GWG (OR 0.93; 95% CI 0.87, 0.99) in women with normal weight.
Yong 2016 [127]	Cross-sectional study	*n* = 589	PA: moderate-vigorous-intensity activity	Pregnant women with low PA level were more likely to have excessive GWG than women with high PA level (OR 1.74; 95% CI 0.77, 3.97).
Harris 2015 [130]	Cross-sectional study	*n* = 856	PA: walking, jogging, aerobics, swimming	Women who engaged in PA at least 3 times a week for 6–9 months during pregnancy had reduced odds of excessive GWG (OR 0.26; 95% CI 0.12, 0.56).
Kraschnewski 2013 [132]	Prospective cohort study	*n* = 2767	PA: minutes per day engaging in regular PA	Pregnant women who engaged in ≥150 min/week had reduced odds of excessive GWG (OR 0.71; 95% CI 0.57, 0.88) compared with inactive women (<60 min/week).
Stuebe 2009 [118]	Prospective cohort study	*n* = 1388	PA: light-to-moderate activities, vigorous activity, total activity (minutes per week)	Vigorous activity, walking and total activity during pregnancy were associated with reduced GWG. 30 min walking/day (OR 0.92; 95% CI 0.83, 1.01), 30 min vigorous PA/day (OR 0.76; 95% CI 0.60–0.97) and 30 min total PA/day (OR 0.95; 95% CI 0.89, 1.01) were associated with lower excessive GWG during mid-pregnancy.
Ehrlich 2016 [128]	Prospective cohort study	*n* = 1055	PA: moderate-intensity and vigorous-intensity	Vigorous-intensity exercise was associated with reduced odds of excessive GWG (OR 0.63; 95% CI 0.40–0.99) overall and in women with overweight/obesity (BMI ≥ 25.0 kg/m^2^) (OR 0.46; 95% CI 0.27, 0.79) but no associations were reported for moderate-intensity exercise.
Jiang 2012 [131]	Prospective cohort study	*n* = 862	PA: daily pedometer step counts	Active pregnant women (≥10,000 daily steps) had lower odds of excessive GWG (OR 0.60; 95% CI 0.35, 1.03) than sedentary women (<5000 daily steps) in the last two trimesters.

‡ Women with singleton pregnancies; Abbreviations: BMI = Body Mass Index; CI = confidence interval; GWG = Gestational weight gain; MD = Mean difference; NND = New Nordic Diet score; OR = odd ratio; PA = Physical activity; RCT = Randomized controlled trial; SD = standard deviation of intake.

### 2.3. Diet, Physical Acitvity and Weight Gain during Preconception and Postpartum

Observational studies assessing associations of diet and PA in preconception and postpartum with weight gain are lacking. Here, preconception is defined as the period in the weeks to months/years before pregnancy for women with a conscious intention or desire to conceive [136] and postpartum is the time period after delivery up to 2 years.

We searched for papers using the databases: Ovid Medline, EMBASE, CINAHL Plus, PsycINFO and all EBM reviews and using the following search terms: “weight gain/or weight adj2 (gain or change) or bmi adj2 (gain or change) or (body mass index) adj2 (gain or change) AND diet/or diet therapy/or diet* or nutri*/or/life$style or lifestyle/or food/or physical activity or exercise AND post$partum or pre$concept* or pre$pregn* or pre$gestation* or peri$conception* or inter$concep* or inter$pregnan* or inter$natal or pre-pregnancy/or postpartum period/or postpartum women. Papers published from 2000 onwards were included if they focused on diet and/or PA and weight gain during preconception and postpartum. Only studies published in English were included.

Only one longitudinal study was identified in preconception. This explored dietary patterns in preconception, pregnancy, postpartum and their association with BMI in 80 Spanish women [71]. This study identified the ‘sweetened beverages and sugars’ pattern from preconception to 6 months postpartum and ‘vegetables and meat’ pattern to the end of pregnancy. The ‘vegetables and meat’ pattern was inversely associated with BMI during the preconception period [71]. However, this study did not specifically assess weight gain during the preconception period. Similarly, there are limited studies assessing the association between diet and PA with weight gain during the postpartum period. To date, only one study specifically assessed postpartum weight gain (weight change from 1 to 6 months postpartum) and diet. This recent longitudinal cohort study in 99 women reported that consumption of added sugars, soft drinks, SSBs and high glycemic diets were associated with weight gain in the first 6 months postpartum [137]. The study reported that a half-serving per day (8 fluid ounces (236.6 mL)) increase of all soft drinks (1.5 kg; (95% CI 0.70, 2.34), increased intake of added sugar (g/day) (0.05 kg; 95% CI 0.004, 0.10), a half 8-ounce serving per day increase in SSBs (0.69 kg; 95% CI 0.06, 1.32), a high glycemic index (bread as comparator) (0.25 kg; 95% CI 0.07, 0.42) and glycemic load (bread comparator) (0.04 kg; 95% CI 0.002, 0.08) were associated with postpartum weight gain, whereas a 1 g/day increment of soluble fiber intake was associated with a decrease in postpartum weight gain (−0.8 kg; −1.35, −0.29) [137].

In contrast, there are several observational studies investigating the association between postpartum weight retention and diet including total energy [138], dietary patterns [72,139,140] or macronutrients (total fat, carbohydrate, protein, saturated fat, and trans-fat and the glycemic index) [141,142], fried food and SSB intake [143,144]. The finding of another recent study also reported that lower diet quality was associated independently with postpartum weight retention despite no association reported in prior studies [72,145]. Total energy intake, regardless of diet composition [72], trans fat intake [141] and discretionary foods [146], has also been reported to be associated with postpartum weight retention. Predictors of postpartum retention including diet and physical activity has been extensively discussed in recent reviews [147].

Finally, there is insufficient evidence on PA and weight gain during the postpartum period or postpartum weight loss [148]. A prospective cohort study (*n* = 1617) reported that total PA (>163.3 MET-hour/week) was inversely associated with weight retention (−0.50 kg; 95% CI −0.94, −0.07) and (−0.66 kg 95% CI −1.09, −0.23) at 6 and 12 months postpartum, respectively, compared to lower total PA (≤133.2 MET-hour/week) [149]. Of those women who were physically active before pregnancy, more than 50% remained sedentary during the postpartum period which may contribute to postpartum weight gain or retention [150]. However, Most et al.’s [138] prospective cohort study of 37 women with obesity found no association of PA or diet quality but a positive association of energy intake with postpartum weight retention. They concluded that weight gain or weight retention during the postpartum period was the result of increased energy intake rather than decreased energy expenditure in women with obesity [138].

In summary, despite the importance of understanding the key contributions of diet and PA in preconception and postpartum to weight gain, there is a lack of research assessing this specific reproductive life stage and a lack of clarity on specific dietary and PA needs to target for the prevention of weight gain through lifestyle modification. This emphasizes the need for observational studies exploring diet and PA components associated with weight gain in preconception and postpartum.

## 3. Interventions for Preventing Weight Gain in Women

### 3.1. Weight Gain Prevention in Reproductive-Age Women

Several studies have been published investigating lifestyle interventions targeting the association of diet and PA with weight gain prevention in women of reproductive age. A randomized controlled trial (RCT) reported that interventions focusing on healthy eating and PA in 622 low-income mothers with overweight/obesity aged 18–34 years were not effective in preventing further weight gain [151]. Metzgar et al. [152] also conducted a RCT which aimed to prevent weight gain in women aged 18–45 years with a BMI of >18.5 kg/m^2^ through nutrition and PA education (emphasizing general nutrition, vegetable consumption, portion control, breakfast consumption, healthy snacking and beverage choices, nutrient density, family menu planning, grocery shopping and PA). This comprised a 12-month intervention with a one-hour nutrition session delivered by a registered dietitian or counsellor weekly for the first 4 months followed by monthly sessions. No significant difference was found between women who received the weight gain prevention intervention and those randomized to a control group. Similarly, Levine and colleagues [153] found no significant effect on weight gain in normal weight and overweight women who received 15 group education sessions or 15 correspondence education lessons compared with an information-only control over three years. Both interventions focused on self-monitoring of energy intake and expenditure and changes in dietary intake such as the substitution of low-fat foods for high-fat and provided a 12-month gym membership [153].

In contrast, Bennett et al. [154] evaluated the effectiveness of weight gain prevention interventions in overweight women aged 25 to 44 years in a rural community health center setting. The intervention was based on the energy deficit approach with tailored behavior change goals (e.g., replacing energy-dense foods with five or more fruits and vegetables/day, no fast food or SSBs) and behavioral change strategies which were reinforced by monthly counselling calls by a trained registered dietitian. Women in the intervention group reduced weight gain compared to usual care (−1.0 vs. 0.5 kg, mean difference between groups −1.4 kg (95% CI −2.8, −0.1)). A 12 months pragmatic cluster randomized controlled trial in 492 women aged 18–50 years by Lombard and colleagues [155] compared women in the control group (receiving one general women’s health education session) with those receiving self-management lifestyle intervention (HeLP-her) through a group session, monthly SMS text messages, one phone coaching session, and a program manual. The intervention was effective in preventing weight gain (−0.5 kg; 95% CI −0.99, 0.03) compared to control group (0.4 kg; 95% CI −0.09, 0.97). A similar study also reported the effectiveness of a low-intensity self-management intervention (simple health messages, behavior change strategies, 3 group sessions and monthly support using mobile telephone text messages for 12 months) in preventing weight gain compared to control (non-interactive information session based on population dietary and PA guidelines) in 250 women aged 25–51 years with young children [156]. In a recent meta-analysis of lifestyle intervention trials targeting adults aged 18–50 years, subgroup analysis by gender (17 studies) showed significant weight reduction following lifestyle interventions compared to controls in women aged 18–50 years (MD −0.92 kg; 95% CI −1.49, −0.36) [39].

In summary, although there are mixed findings in relation to the success of lifestyle interventions targeting diet and PA to prevent weight gain overall, there is evidence of efficacy of diet and PA interventions for weight gain preventions in women of reproductive age. This suggests that we can support women’s health during these important life years.

### 3.2. Prevention of Excessive Gestational Weight Gain during Pregnancy

Previous systematic reviews and meta-analyses have reported efficacy of diet and/or PA interventions to reduce excessive GWG [157,158,159,160,161,162,163,164]. The international weight management in pregnancy (iWIP) [161] collaborative group synthesized individual participant data from 36 randomized trials and documented that diet and PA based interventions in pregnancy reduced GWG (MD −0.70 kg; 95% CI −0.92, −0.48) [161]. A systematic review by Thangaratinam et al. [164] that included 44 RCTs (7278 women) and evaluated diet and/or PA interventions reported a 1.42 kg (95% CI 0.95, 1.89) reduction in GWG with any intervention compared with control. Diet only interventions were most effective (MD −3.84 kg; 95% CI −5.22, −2.45) in reducing GWG than PA (MD −0.72 kg; 95% CI −1.20, −0.25) or combined interventions (MD −1.06; 95% CI −1.67, −0.46). Here, diet only interventions included balanced diets of 18–24 kJ/kg with higher intake of fruits, vegetables and legumes; healthy diets with a maximum of 30% fat, 15–20% protein, and 50–55% carbohydrate or low glycemic diets with unprocessed whole grains and energy intake individualized to the needs of pregnant women. Subgroup analysis by BMI showed a reduction in GWG in all BMI classes (MD −5.53 kg; 95% CI −8.54, −2.53) and women with overweight/obesity (MD −7.73 kg; 95% CI −9.40, −6.05 kg). The PA interventions included light intensity resistance training, weight-bearing exercises and walking for 30 min. The LIFE-Moms consortium [165] conducted several RCTs in pregnant women with overweight and obesity to evaluate the effectiveness of lifestyle interventions in limiting excessive GWG. A prospective meta-analysis of lifestyle intervention trials targeting 1150 women with overweight and obesity showed significant reduction in excessive GWG compared to standard care (MD −1.59 kg; 95% CI −2.18, −0.99) [166].

In general, there is sufficient evidence on successful lifestyle interventions to prevent excess GWG during pregnancy although there is a variation in lifestyle intervention or specific details of the diet and PA components needed for effective interventions. A recent comprehensive review of reviews recommended multi-component lifestyle interventions including a balanced diet with low glycemic index foods and light to moderate-intensity PA 30–60 min per day 3–5 days per week to prevent excessive weight gain during pregnancy [167].

### 3.3. Weight Gain Prevention in Preconception and Postpartum

There is a paucity of research on lifestyle interventions (diet and/or PA) aimed at the prevention of weight gain in preconception. Interventions in the preconception period have focused on improving general nutritional status and health behaviors aimed at improving reproductive health and pregnancy outcomes [168]. While these may indirectly benefit weight, they are generally not directly targeted at preventing weight gain. Despite the lack of literature specific to preconception, there is evidence on effectiveness of lifestyle interventions in preventing weight gain in reproductive-age women, which can be adopted for women in the preconception period. Although the preconception period can be a “teachable moment” for lifestyle modification, opportunities are often missed as nearly half of pregnancies are unplanned [169]. Furthermore, women preconception face several barriers to adopting a healthy lifestyle and weight including lack of knowledge, not planning pregnancy, beliefs about negative consequences of eating healthy food, family pressure and lack of resources [170].

Lifestyle intervention studies targeting weight gain prevention postpartum are also lacking with most of the previous observational studies, trials and systematic reviews and meta-analysis assessing dietary and PA postpartum focusing on reduction of postpartum weight retention [157,171,172,173,174,175,176]. To our knowledge, no studies have assessed the effect of lifestyle (diet and/or PA) interventions specifically on preventing weight gain postpartum. A recent review of 17 studies assessed dietary changes from pregnancy to postpartum and reported that postpartum women are more likely to have a higher intake of discretionary choices, decreased fruit and vegetable consumption and poor adherence to a healthier dietary pattern during the transition from pregnancy to postpartum [177]. As previously discussed in reproductive-age women overall, these diet components and patterns would likely contribute to further weight gain postpartum. Lifestyle interventions targeting key components such as limiting energy intake from discretionary choices; increasing consumption of core foods including fruits, vegetables and whole grains and engaging in regular PA may therefore aid in preventing weight gain in the postpartum period. Postpartum women also experience many barriers to attaining a healthy lifestyle such as increased family and time commitments, limited time, lack of motivation and confidence, fatigue and lack of access to appropriate and affordable exercise facilities [178]. Weight gain prevention interventions to support women in the postpartum period should also consider these unique barriers. The postpartum period can also be considered as inter-conception if another pregnancy occurs.

Overall, we summarize here a large body of observational and population-based studies assessing the contribution of diet and PA to weight gain in reproductive age women. While these studies provide important insights with regard to which diet and PA components to target, they are limited with regard to inferring causal mechanisms which should be a focus of future research. Furthermore, observational studies can be influenced by confounding and here often relied on often on self-reported dietary intake and PA which can be subject to recall bias. Similarly, several systematic reviews and meta-analyses of RCTs strongly suggest the effectiveness of lifestyle (diet and/or PA) in reducing weight gain in women of reproductive age and preventing excessive GWG during pregnancy. However, there is a lack of clarity on diet and PA components to be targeted during interventions which need to be addressed in future research. The role of intake of micronutrients with weight gain and weight gain prevention was not addressed in this review. Evidence on the importance of micronutrients and overweight/obesity is evolving and future studies should investigate the effect of micronutrients, nutrient interactions and optimal intake during preconception, pregnancy and postpartum and weight gain in reproductive life stages.

PA is inversely associated with weight and plays a pivotal role in weight gain prevention. Most of the studies included in this review used self-report measures which may overestimate PA. Use of objective measures of PA such as instruments or devices including accelerometers would give less biased results and a more accurate reflection of the amount or type of PA required to prevent weight gain including aspects such as intensity, duration, frequency or time of day. Further studies are required to document associations of objectively measured PA levels with changes in weight.

## 4. Summary

This comprehensive review summarizes the existing evidence on the relationship between specific diet and PA components with weight gain in women of reproductive age and key reproductive life stages (preconception, pregnancy and postpartum). Furthermore, the review provides an overarching summary of lifestyle intervention studies, primarily RCTs, aimed at preventing weight gain in these population groups. There is clear evidence of a positive association between high energy intake from added sugars, confectionary, saturated fat and processed foods with weight gain and an inverse association between increased consumption of fruits, vegetables and whole grains with weight gain in women of reproductive age. However, further research is required to more clearly understand the effect of these diet components on weight gain in women in key reproductive life stages. It is also evident that increasing PA is inversely associated with weight gain. However, the specific level of PA required to prevent weight gain remains unclear, especially in reproductive life stages. The existing evidence also suggests that lifestyle (diet and/or PA) interventions are effective in reducing weight gain in women of reproductive age and preventing excessive GWG during pregnancy. However, evidence on diet and PA components contributing to weight gain and lifestyle interventions to prevent weight gain specifically during preconception and during postpartum are lacking. There is therefore a need for further research to identify key dietary and PA components targeted at specific reproductive life stages to guide intervention strategies in preventing weight gain. There is also a need for further research to optimize dietary intake and physical activity to ensure optimal energy balance in key reproductive life stages.
